# Day–night fluctuations in choroid plexus transcriptomics and cerebrospinal fluid metabolomics

**DOI:** 10.1093/pnasnexus/pgad262

**Published:** 2023-08-10

**Authors:** Beatriche Louise Edelbo, Søren Norge Andreassen, Annette Buur Steffensen, Nanna MacAulay

**Affiliations:** Department of Neuroscience, University of Copenhagen, 2200 Copenhagen, Denmark; Department of Neuroscience, University of Copenhagen, 2200 Copenhagen, Denmark; Department of Neuroscience, University of Copenhagen, 2200 Copenhagen, Denmark; Department of Neuroscience, University of Copenhagen, 2200 Copenhagen, Denmark

**Keywords:** CSF, sleep, circadian rhythm, metabolomics, transcriptomics

## Abstract

The cerebrospinal fluid (CSF) provides mechanical protection for the brain and serves as a brain dispersion route for nutrients, hormones, and metabolic waste. The CSF secretion rate is elevated in the dark phase in both humans and rats, which could support the CSF flow along the paravascular spaces that may be implicated in waste clearance. The similar diurnal CSF dynamics pattern observed in the day-active human and the nocturnal rat suggests a circadian regulation of this physiological variable, rather than sleep itself. To obtain a catalog of potential molecular drivers that could provide the day–night-associated modulation of the CSF secretion rate, we determined the diurnal fluctuation in the rat choroid plexus transcriptomic profile with RNA-seq and in the CSF metabolomics with ultraperformance liquid chromatography combined with mass spectrometry. We detected significant fluctuation of 19 CSF metabolites and differential expression of 2,778 choroid plexus genes between the light and the dark phase, the latter of which encompassed circadian rhythm–related genes and several choroid plexus transport mechanisms. The fluctuating components were organized with joint pathway analysis, of which several pathways demonstrated diurnal regulation. Our results illustrate substantial transcriptional and metabolic light–dark phase–mediated changes taking place in the rat choroid plexus and its encircling CSF. The combined data provide directions toward future identification of the molecular pathways governing the fluctuation of this physiological process and could potentially be harnessed to modulate the CSF dynamics in pathology.

**Significance Statement**

The cerebrospinal fluid (CSF) secretion rate and the associated intracranial pressure are elevated in the dark phase in both humans and rats, irrespective of sleep pattern. Potential molecular drivers may be found within the catalog of fluctuating choroid plexus transcripts and metabolites of the surrounding CSF. Both of these components demonstrate substantial diurnal regulation, which, on their own or jointly, could contribute to the light–dark phase–mediated modulation of the CSF dynamics.

## Introduction

Cerebrospinal fluid (CSF) fills the ventricular spaces and encircles the mammalian brain and spinal cord, providing protection against mechanical insult, and serves as a dispersion route for nutrients, metabolic waste, and signaling molecules between various brain cells and structures. Aberrant accumulation of CSF is observed in connection with a number of cerebral pathologies, i.e. congenital or secondary hydrocephalus and stroke-induced cerebral edema, which may associate with life-threatening elevation of the intracranial pressure (ICP) if left untreated. Standard care of such conditions relies on invasive neurosurgical procedures including ventriculoperitoneal shunt insertion, which, although lifesaving, are hampered by frequent shunt failures, infections, and other complications (1, 2). Efficient and specific pharmacological treatment of conditions involving elevated ICP is lacking, in part due to our limited understanding of the molecular mechanisms underlying CSF dynamics and the regulation thereof.

The majority of the CSF is produced by the choroid plexus, which is a specialized epithelium localized in each of the four ventricles (3). The choroid plexus expresses a variety of membrane transport mechanisms (4), a concerted effort of which underlies the fluid secretion across this epithelium (5–7). The CSF secretion rate appears to be diurnally regulated, with an elevation in the dark phase in both humans and rats (8, 9), which may underlie the dark phase–related elevation in ICP detected in both species (9, 10). The similar diurnal regulation of the CSF dynamics observed in the diurnal human and the nocturnal rat suggests a circadian regulation of this physiological variable, rather than sleep itself (9), as was also observed for the CSF flow along the paravascular spaces into the brain tissue (11), hypothesized to support waste clearance in the rodent brain (12, 13). However, the molecular drivers of the diurnal fluctuation in CSF secretion remain unresolved but could potentially be found in either the choroid plexus tissue itself and/or in the CSF surrounding it.

Here, we determine the light–dark phase fluctuations in the rat choroid plexus transcriptome and in the rat CSF metabolome to create a catalog of potential regulatory properties of these compartments that could contribute to the circadian modulation of the CSF secretion rate.

## Results

### Metabolomics profile of rat CSF

**Fig. 1.**
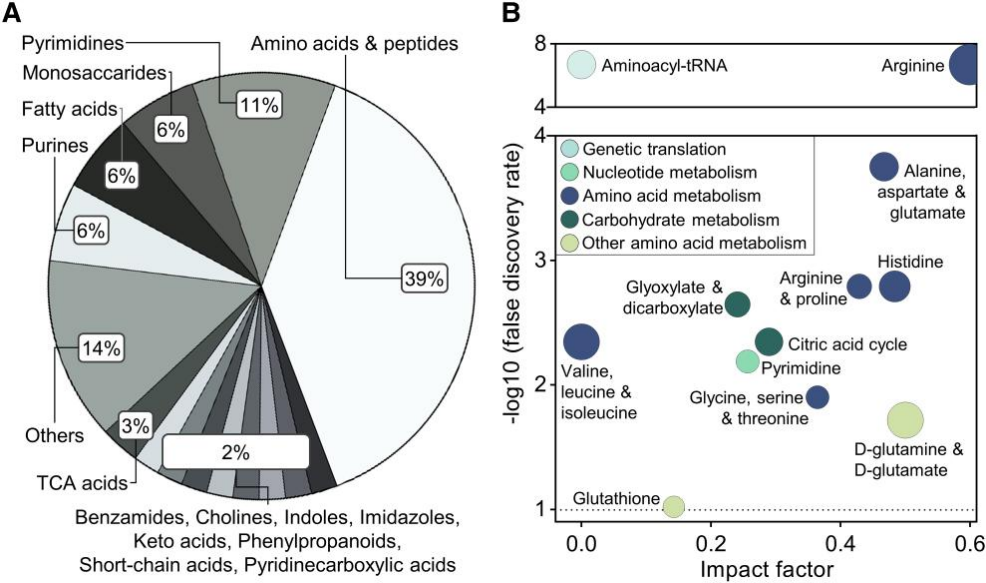
CSF metabolomics. A) Distribution of main compound classes detected in the rat CSF metabolome. The section “Others” encompasses the 18 categories represented by only one metabolite (see Table S1). B) Pathway analysis of the CSF metabolome. Pathways are organized according to the collected impact of the detected pathway-specific metabolites (*x*-axis) against the probability of the metabolite composition being a function of that particular pathway (*y*-axis). Spheres are arranged according to the subclass of pathways (see inset); sphere area indicates the number of metabolites detected compared to the expected number (fold enrichment) (Table S2). The dashed line indicates cutoff for pathway significance (at a FDR < 0.10). Data are based on CSF collected from 23 rats (*n* = 12 rats in the dark phase and n = 11 rats in the light phase).

To determine the metabolite composition of rat CSF, this fluid was extracted from anesthetized rats and analyzed using liquid chromatography mass spectrometry (LC-MS). One hundred twenty-six different metabolites were identified in the CSF (Table S1) and were categorized into main classes with enrichment analysis (MetaboAnalyst) (Fig. 1A). The largest class comprised amino acids and peptides (39%), followed by pyrimidines (11%), purines (6%), monosaccharides (6%), fatty acids (6%), tricyclic acids (3%), and smaller categories (<3%) of indoles, imidazoles, cholines, in addition to various acids, benzamides, and others (Fig. 1A; Table S1). Of the 86 rat CSF metabolites that satisfied inclusion criteria (see Materials and methods), the majority (57 metabolites; 66%) matched those of human CSF metabolites extracted from Human Metabolome Database (HMDB) (Fig. S1). Of the remaining rat CSF metabolites, several are metabolic precursors (or successors) to metabolites registered as present in human CSF, e.g. 5-methoxytryptophan, *N*-acetyl-tyrosine, and *N*-acetyl-glutamine (Table S1).

To determine whether the detected metabolites were arranged into relevant biological pathways, these were grouped by pathway analysis (see Materials and methods). Pathway analysis assesses the biological relevance of pathways by identifying the collected impact of pathway-specific metabolites detected, against the probability of the metabolite composition observed being a function of that particular pathway. The biological pathways detected in the rat CSF were mainly related to amino acid metabolism (6/12), with the remaining pathways belonging to either carbohydrate metabolism, metabolism of other amino acids, genetic translation, or nucleotide metabolism (Fig. 1B; Table S2). Most notably, the pathway underlying arginine biosynthesis was highly significant and presented with a high impact in the CSF, alongside several other amino acid metabolism pathways. In addition, two pathways involved in energy conversion through carbohydrate metabolism; the citric acid cycle and glyoxylate and dicarboxylate metabolism were also present in the CSF (Fig. 1B).

### CSF metabolites fluctuate with the light–dark phase cycle

**Fig. 2.**
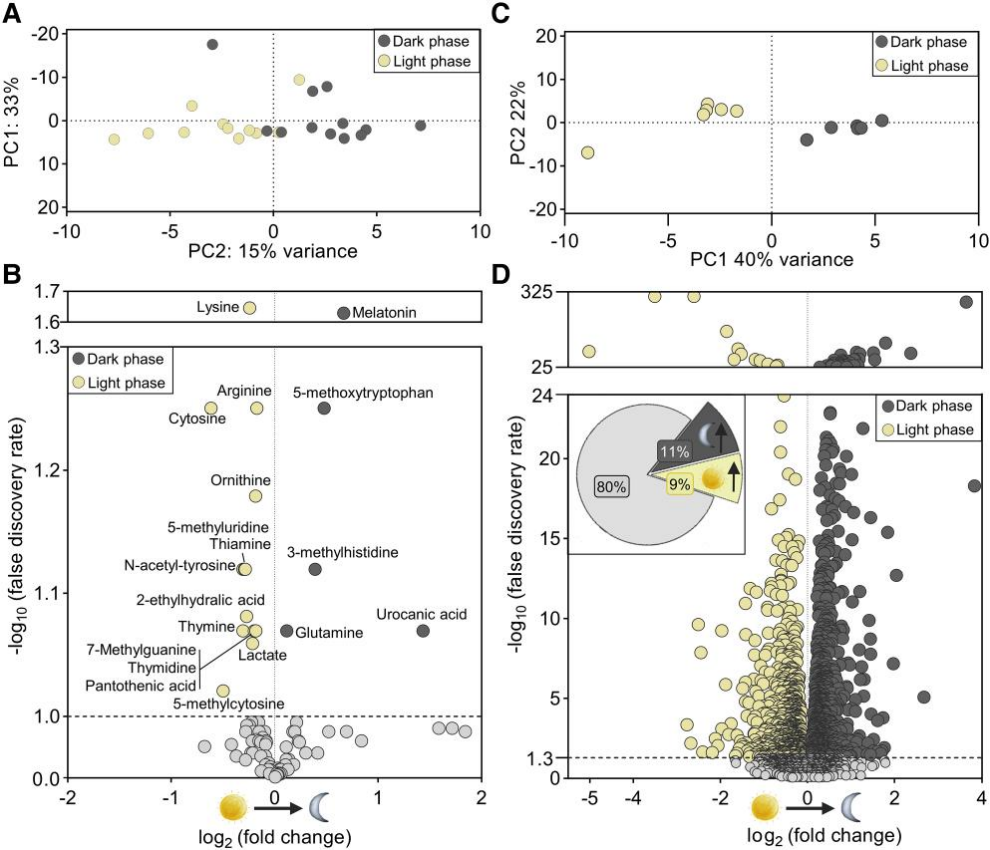
Diurnal modulation of the CSF metabolome and the choroid plexus transcriptome. A) The normalized abundance of rat CSF metabolites obtained during the dark and the light phase were plotted with respect to their first and second principle component (PC1 and PC2). B) Volcano plot of CSF metabolites identified with the fold change (log_2_-transformed) between light and dark phases. Color coding (see inset) denotes metabolites significantly more abundant during either phase. The dashed line indicates cutoff for significance (at a FDR < 0.10). Data are based on CSF collected from *n* = 12 rats in the dark phase and *n* = 11 rats in the light phase. C) DEseq2 normalized values for genes expressed in the rat choroid plexus excised during the dark and the light phase were plotted with respect to their first and second principal component (PC1 and PC2). D) Volcano plot of genes expressed by the choroid plexus in the dark and the light phase with the fold change (log_2_-transformed) on the *x*-axis. The color coding denotes gene transcripts significantly up-regulated during either phase. The inset illustrates the distribution of differentially expressed genes and colored based on up-regulation within the light (yellow) and dark (dark gray) phase with the non-differentially expressed genes marked in light gray. The dashed line indicates cutoff for significance (at a FDR < 0.05). Data are based on choroid plexuses extracted from *n* = 6 rats in the dark phase and *n* = 6 rats in the light phase.

To determine whether the rat CSF metabolome fluctuated with the light–dark phase cycle, we compared the metabolite composition in CSF extracted from anesthetized rats in the peak light phase (8 h after light phase initiation, zeitgeber time 8 h) with CSF extracted from anesthetized rats in the peak dark phase (8 h after dark phase initiation, zeitgeber time 20 h, with care taken to shield the rats from light during the anesthesia and CSF extraction procedure). A principal component analysis (PCA) plot of the tested metabolites demonstrated that the CSF extracted at these two time points separated into two groups (Fig. 2A), suggesting an overall diurnal difference in CSF metabolite composition. A volcano plot of the light–dark phase metabolite presence illustrated that 19 (out of 86 included metabolites; 22%) were detected at significantly different levels in the light and the dark phase, with five metabolites (6%) increased in the dark phase and 13 (15%) increased in the light phase (Fig. 2B; Table S1). Among the metabolites increased in the dark phase were melatonin, urocanic acid, and 5-methoxytryptophan (a precursor of several biologically active molecules including melatonin and serotonin), while lysine, cytosine, arginine, and 5-methylcytosine, among others, were increased in the light phase (Fig. 2B). To determine whether this diurnal fluctuation in the metabolite composition was reflected in the biological pathways represented in the CSF, the pathway analysis was conducted with inclusion of the fold change metabolite presence between light phase and dark phase (Fig. S2 and Table S3). Eight of the originally identified pathways present in the CSF contained diurnally fluctuating metabolites (Fig. S2), among which we detected arginine biosynthesis and several other pathways involved in amino acid metabolism. Notably, many of the pathways presented with high significance but low impact factor, or vice-versa, indicating that the diurnal change in metabolite composition may, rather, be achieved in combination with other processes occurring within the brain tissue.

### Choroid plexus gene expression differs with the light–dark phase cycle

**Fig. 3.**
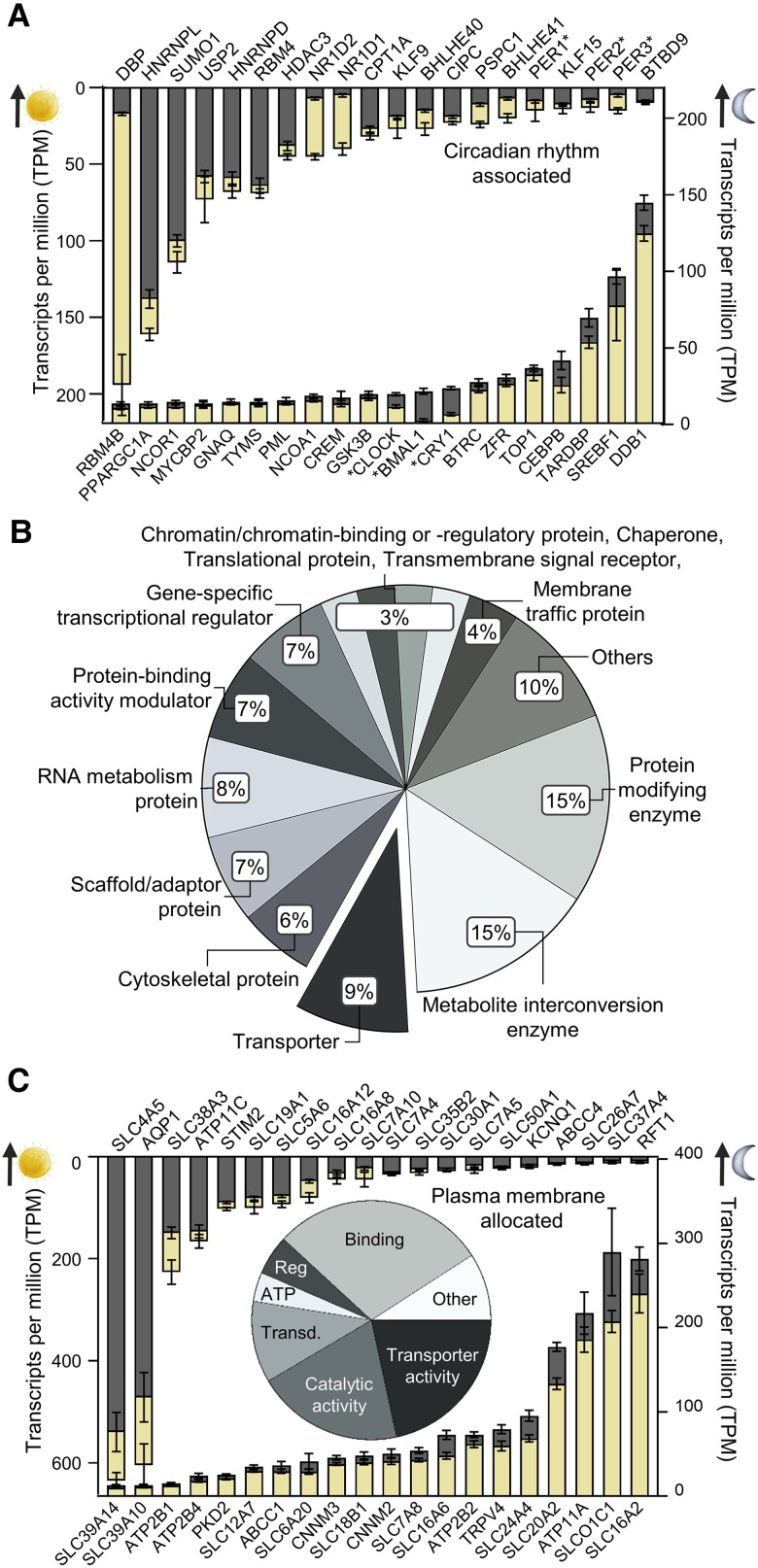
Characterization of the diurnally regulated choroid plexus transcriptome and transportome. A) Choroid plexus gene expression of the 20 circadian rhythm–associated genes with the most pronounced shifts between the dark–light phases. Gene names denoted with * are CLOCK genes. Data are based on *n* = 6 and are shown as mean ± SD, and all genes had a TPM sum above 20. Dark phase gene expression is illustrated in gray with light phase gene expression illustrated in yellow. B) The dark–light phase differentially expressed genes in the rat choroid plexus were distributed according to protein classes, based on GO enrichment analysis of differentially expressed genes. C) Choroid plexus gene expression of the 20 plasma membrane–associated transporter genes with the most pronounced shifts between the dark–light phases. Data are based on *n* = 6 and are shown as mean ± SD, and all genes had a TPM sum above 20. Dark phase gene expression is illustrated in gray with light phase gene expression illustrated in yellow. Inset, the molecular function of differentially expressed genes. Categories amounting to 2% or less are collected in “Other.” ATP, ATP-dependent activity; Reg, molecular function regulator; Transd., molecular transducer activity.

To determine whether the choroid plexus transcriptomic profile fluctuates with the light–dark cycle and thus may underlie the diurnal regulation of CSF secretion, we performed RNA-seq on choroid plexus acutely excised from rats in the peak light phase (zeitgeber time 8 h) and from rats in the peak dark phase (zeitgeber time 20 h) (Table S4). A PCA plot demonstrated that the overall transcriptomic profile of the choroid plexus differed between the two phases (Fig. 2C). Differential expression analysis revealed that of the approximately 14,000 genes expressed above the threshold in the rat choroid plexus (see Materials and methods), 2,778 genes (20%) were differentially expressed, with 1,496 genes (11%) up-regulated in the dark phase and 1,282 genes (9%) up-regulated in the light phase (Fig. 2D inset; Table S5). A volcano plot demonstrated up to a 30-fold difference (5 log_2_-fold change) in the diurnally modulated transcript fluctuations (Fig. 2D). Among the 20 gene transcripts with the largest increase in either phase (Tables S6 and S7), we find three CLOCK genes (PER1, BMAL1, and CRY1) and genes involved in transcription and translation. Of all the transcripts detected in choroid plexus, 278 were associated with “circadian rhythm,” with approximately 30% (81 genes) fluctuating with the dark–light phase (Table S8). Figure 3A illustrates the expression level of the 20 transcripts encoding circadian rhythm–associated genes (with a combined light–dark phase transcripts per million [TPM] sum ≥ 20) with the largest fluctuations. Notably, six of the 10 CLOCK genes were present among the 20 gene transcripts with the largest fluctuations (Fig. 3A). The data demonstrate light–dark phase modulation of choroid plexus gene transcription, and thus function, and suggest that the dark–light phase–mediated changes in choroid plexus function could occur via circadian rhythm–regulated genes and various modulatory genes potentially permissive for a range of regulatory actions on diverse choroid plexus proteins.

### Choroid plexus transporter transcripts fluctuate with the light–dark phase cycle

To assess which protein classes were affected by the diurnal changes in transcription, gene ontology (GO) enrichment analysis was performed on the differentially expressed genes with exclusion of the 902 genes (32% of the detected differentially expressed genes) that did not have a protein class assigned. Eleven different protein classes each contained ≤2% of the transcripts and were combined in “Others” (intercellular signal molecule, DNA metabolism protein, cell adhesion molecule, extracellular matrix protein, defense/immunity protein, transfer/carrier protein, structural protein, calcium-binding protein, cell junction protein, viral or transposable element protein, and storage protein). The majority of the differentially expressed genes encode metabolite interconversion enzymes (15%) and protein-modifying enzymes (15%), followed by the category comprising transporters (9%) and various other smaller categories (Fig. 3B). To determine whether the dark–light phase modulation of CSF dynamics (9) could be attributed to dark–light phase–induced differential expression of choroid plexus plasma membrane transporters, differentially expressed genes assigned to the plasma membrane fraction were initially extracted with compartment analysis (Tables S9 and S10). Of these dark–light phase–modulated genes, the largest fraction comprised genes involved in “binding” (i.e. receptors, signaling molecules, and scaffold proteins, 21%), followed by the transporter fraction (15%), transcripts involved in catalytic activity (14%), and various other functions (Fig. 3C, inset). Figure 3C illustrates the expression level of the 20 transcripts encoding plasma membrane transporters (with a combined light–dark phase TPM sum ≥ 20) with the largest fluctuations. Among the 20 most up-regulated plasma membrane–associated genes in the light phase were the electrogenic Na^+^,HCO_3_^−^ cotransporter 2 NBCe2 (*SLC4A5*, 18%), the water channel aquaporin 1 (*AQP1*, 28%), the sodium-coupled neutral amino acid transporter 3 SNAT3 (*SLC38a3*, 51%), and the folate transporter 1 FOLT (*SLC19A1*, 23%). Among the transporter genes that were most up-regulated in the dark phase, we detected the monocarboxylate transporter MCT6 (*SLC16A2*, 17%), the organic anion transporter OATP1 (*SLCO1C1*, 40%), the Na^+^-dependent phosphate transporter 2 PIT2 (*SLC20A2*, 33%), the potassium-dependent sodium/calcium exchanger NCX4 (*SLC24A4*, 39%), and the transient receptor potential vanilloid 4 ion channel TRPV4 (*TRPV4*, 33%). These data could suggest that diurnal regulation of choroid plexus transporters could contribute to the fluctuating CSF secretion rate. However, with the relatively modest numerical changes, we speculated whether differences in choroid plexus metabolic activity could modulate the energy-dependent transporters supporting the CSF secretion and, thus, in addition, determined the diurnal fluctuation in transcripts encoding mitochondrial genes (Tables S9 and S11). Among the differentially expressed genes, those assigned to “catalytic activity” by GO enrichment analysis of biological function were highest represented (37%), followed by those assigned to “binding” (16%), ATP-dependent activity (4%), transporter activity (4%), and other smaller groupings (Fig. S3A, inset). Even among the 20 most differentially expressed genes, the numerical differences between the dark and the light phase were minor (Fig. S3A), and diurnal fluctuations in mitochondrial genes were therefore not expected to underlie the dark phase increase in CSF secretion. Of the metabolite interconversion enzymes, the largest fraction comprised genes involved in catalytic activity (83%) followed by “binding” (i.e. receptors, signaling molecules, and scaffold proteins, 13%), ATP-dependent activity (2%), and various other functions (2%) (Fig. S3B, inset). Among the 20 gene transcripts with the largest increase in either phase, two genes involved in different metabolic processes displayed the most prominent diurnal fluctuation; peroxiredoxin 1 (*PRDX1*, 30% up-regulation in the light phase) and adenosylhomocysteinase like 2 (*AHCYL2*, 44% up-regulation in the dark phase) (Fig. S3B).

### Joint pathway analysis of light–dark phase cycle–modulated metabolic pathways

**Fig. 4.**
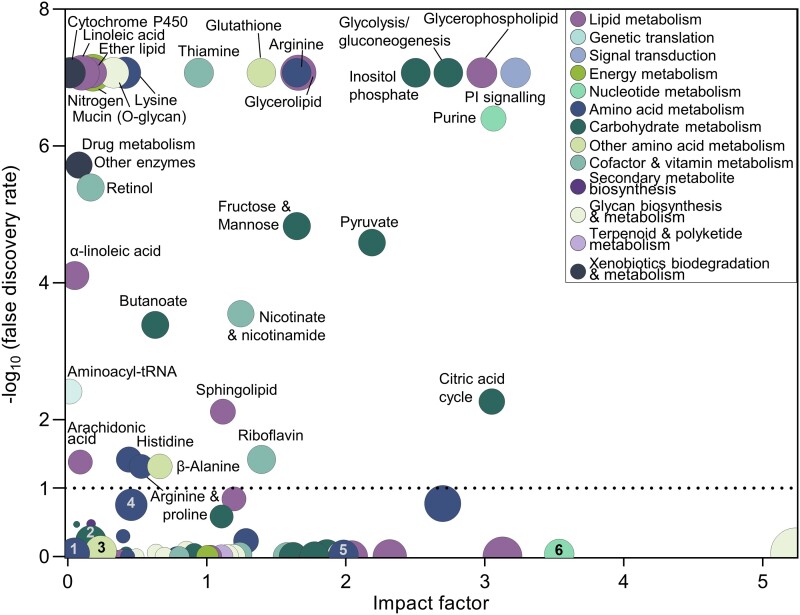
Joint pathway analysis of diurnally modulated CSF metabolome and choroid plexus transcriptome. Biological pathways identified by combining the CSF metabolome with the choroid plexus transcriptome, with the inclusion of fold change between light and dark phases. Pathways are organized according to the collected impact of pathway-specific metabolites detected (*x*-axis) against the probability of the metabolite composition being a function of that particular pathway (*y*-axis). The sphere color specifies the subclass of pathways (see inset); sphere area represents the number of metabolites detected compared with the expected number (fold enrichment) (see Table S12). The dashed line indicates cutoff for pathway significance (at a FDR < 0.10). The biological pathways that were significant when exclusively employing the CSF metabolome with diurnal modulation for the analysis (Fig. S2 and Table S3) are numbered (1: valine, leucine, and isoleucine biosynthesis; 2: glyoxylate and dicarboxylate metabolism; 3: d-glutamine and d-glutamate metabolism; 4: alanine, aspartate, and glutamate metabolism; 5: glycine, serine, and threonine metabolism; 6: pyrimidine metabolism) (Table S12).

To connect the diurnal fluctuations in CSF metabolomics with those of the choroid plexus transcriptome, we performed joint pathway analysis of the two data sets. In addition to the 47 pathways previously identified (Table S3), 37 biological pathways emerged from the combined analysis (Table S12). Of these 84 biological pathways, 30 reached significance (false discovery rate [FDR] < 0.10) with pathways underlying carbohydrate and lipid metabolism (inositol phosphate metabolism, citric acid cycle, pyruvate metabolism, glycolysis/gluconeogenesis, and glycerophospholipid metabolism) as well as nucleotide metabolism and signal transduction (purine metabolism and phosphatidylinositol signaling system) presenting with a high impact factor (Fig. 4). Furthermore, six of the previously significantly fluctuating biological pathways (Fig. S2; Table S3) were no longer significant upon inclusion of the transcript analysis (Fig. 4; Table S12). Among these were pathways involving amino acid metabolism (valine, leucine, and isoleucine biosynthesis; alanine, aspartate, and glutamate metabolism; and glycine, serine, and threonine metabolism), carbohydrate metabolism (glyoxylate and dicarboxylate metabolism), other amino acid metabolism (glutamine and glutamate metabolism), and nucleotide metabolism (pyrimidine metabolism). To demonstrate the light–dark phase modulation of the significant CSF biological pathways occurring with inclusion of the choroid plexus transcriptome, we illustrate the *P*-value *change* occurring with the inclusion (Fig. S4). Among the pathways with elevated level of significance, we detected those involving carbohydrate metabolism (inositol phosphate metabolism and glycolysis/gluconeogenesis), lipid metabolism (glycerophospholipid metabolism), and nucleotide metabolism (purine metabolism), indicative of the significance and impact depending on differential expression of choroid plexus genes. With the citric acid cycle and glycolysis/gluconeogenesis presenting with a high impact factor in the joint pathway analysis, we determined the diurnal fluctuation of a range of genes involved in these processes. Notably, several genes involved in the citric acid cycle and glycolysis were up-regulated in choroid plexus isolated in the dark phase (Table S13). Our data suggest that the diurnal fluctuations in CSF metabolite composition and choroid plexus transcriptomics may jointly impact biologically relevant pathways.

## Discussion

Here, we reveal diurnal regulation of the rat CSF metabolomic profile and the choroid plexus transcriptome and propose their implication in the diurnal regulation of CSF dynamics. Our brains contain approximately 80% water, parts of which constitute the CSF that fills the cerebral ventricles and subarachnoid space surrounding the brain, in addition to the interstitial fluid residing between cells and structures within the brain. As such, the CSF serves as the main transport route for nutrients, hormones, signaling molecules, and metabolic waste products (12–14). These processes are aided by the continuous replacement of the CSF following de novo fluid secretion of 500 ml/day from the vasculature in the adult human brain (7), leading to replacement of the CSF and its content a predicted three to four times a day. The interstitial fluid waste removal is proposed to occur at an increased rate with sleep (12, 13), a process governed by the circadian rhythm in mice demonstrating larger CSF tracer influx in anesthetized mice during the light phase (15) or, alternatively, elevated redistribution of the employed contrast agent in awake rats during the dark phase (16). Such processes could be supported by the elevated rate of CSF secretion occurring in the dark phase in both rat and human (8, 9). The dark phase elevation of CSF secretion and ICP occurred in both tested species irrespective of their nocturnal (rat) or diurnal (human) activity level, which suggests that these processes are governed by the circadian rhythm rather than by sleep itself (9) and could, as such, be governed by diurnal fluctuations in choroid plexus gene transcription or following fluctuations in potential regulatory factors present in the CSF.

Transcriptomic analysis performed on RNA extracted from choroid plexus isolated during the light phase and the dark phase demonstrated that 20% of the choroid plexus transcriptome was differentially expressed between the two phases, which amounts to nearly 3,000 gene transcripts fluctuating with time of day, as previously observed in other tissues (17, 18). Approximately half of these were up-regulated in the dark phase, with the other half up-regulated in the light phase, indicating a vast functional change in this tissue with time of day (19). Among the differentially expressed genes were several CLOCK genes (20), with a dark phase up-regulation of Arntl (BMAL1), Cry1, and CLOCK, while Per1 and Per2 were up-regulated in the light phase. This fluctuation indicates an overall up-regulation of the CLOCK/BMAL1 transcription during dark phase, which associates with transcription of CLOCK-controlled genes (21) and aligns with earlier findings of an internal clock in the mouse and rat choroid plexus (19, 22–25). Similar CLOCK-dependent regulation is linked to astrocyte activity supporting neuronal activity and governing behavioral states (26–28). Curiously, even the blood–brain barrier appears to house an internal clock, which controls the efflux transporters and thus affects drug delivery to the central nervous system (29–31). As such, CLOCK-dependent regulation of choroid plexus tissue could lead to fundamental changes in tissue function.

The CSF secretion is supported by membrane transport mechanisms located in the choroid plexus plasma membrane, some of which are well established (3, 6) and some of which may be found in the vast list of other transporters identified in the choroid plexus (4). Dark phase up-regulation of select transport mechanisms could contribute to the elevated CSF secretion in this phase, and extraction of plasma membrane transport proteins from the differentially expressed transcripts revealed MCT6, OATP1, PIT2, and NCX4 as up-regulated (20–40%) in the choroid plexus extracted at night. To our knowledge, none of these transporters have been investigated for their role in CSF secretion, while the up-regulated transient receptor potential vanilloid 4 ion channel TRPV4 (33%) (9) is demonstrated to activate the Na^+^,K^+^,2Cl^−^ cotransporter 1 (32) that is implicated in CSF secretion (33–35). This diurnal modulation of several transporters, potentially along with various kinases, signaling cascades, and other modulatory factors, could underlie part of the observed dark phase increase in CSF secretion (9), possibly in combination with modulatory factors residing in the CSF bathing the choroid plexus tissue.

Metabolomic analysis of CSF revealed a range of metabolites that arranged into various classes, predominantly amino acids and peptides, pyrimidines, purines, monosaccharides, and fatty acids, as previously observed for human CSF (36). The majority of the identified rat CSF metabolites are registered for human CSF in HMDB. Notably, 22 of the remaining 29 metabolites not ascribed to human CSF in HMDB were either identified in human CSF in previous studies (36–38) or known precursors to metabolites identified within human CSF. As such, these metabolites are highly likely to be present within human CSF. Human CSF is, in addition, usually sampled from the lumbar region and not from the ventricular compartment as in the present study, which influences proteomic composition (39) and therefore possibly also metabolite composition. CSF collected in the light phase versus that of the dark phase revealed differential abundance of 22% of the CSF metabolites (19 of the included 86 metabolites). Although the percentage fluctuating metabolites is similar to the 20% day–night fluctuating transcripts of the choroid plexus, one generally does not find as vast changes in the metabolomics profile as in the transcriptome. However, changes in few metabolites can be of high importance for the circadian rhythm (40–42). Melatonin appeared as a prominent dark phase up-regulated metabolite. Melatonin is a key factor in circadian control (43) and is increased in the dark phase of both day-active humans and nocturnally active rats, suggesting that this regulatory hormone evolved for its purpose prior to certain species becoming nocturnal of nature (44, 45). Melatonin levels are inversely proportional to serotonin levels, as serotonin is a precursor for melatonin (46). It follows that serotonin demonstrates diurnal fluctuation, as observed in several species (47, 48). With the proposed serotonin-mediated modulation of the rate of CSF secretion (49, 50), such light–dark phase fluctuations may contribute to the diurnal regulation of CSF secretion. An alternative melatonin synthesizing pathway involves 5-methoxytryptophan (51), which was also significantly increased in the CSF in the dark phase.

Diurnal variation in metabolic profile has earlier been reported for plasma, and both plasma metabolite content and its diurnal variation appear impacted by sleep disturbances (40, 52, 53). Connection of the metabolome into different biological pathways revealed several enriched pathways in the CSF, most prominently those involved in arginine biosynthesis and other amino acid metabolism pathways, i.e. alanine, aspartate, glutamate, arginine, proline, and histidine metabolism, in addition to the citric acid cycle. These components are expected to originate from cells surrounding the ventricles and thus reflect the biological activity of a mixture of cell types. With the addition of light–dark fluctuations, several pathways decreased in impact factor, and/or significance level, suggesting that these pathways are not subject to diurnal regulation. In contrast, pathways such as that involved in arginine biosynthesis demonstrated the opposite, which aligned with dark–light fluctuating levels of arginine, together indicating a diurnal modulation of this amino acid.

With the complexity of biological functions within living organisms, it may be beneficial to combine various aspects of cell function (54), possibly by combination of different “omic” disciplines (55, 56). To decipher a potential interplay between the diurnally regulated choroid plexus transcripts and the metabolic pathways in the surrounding CSF, we combined CSF metabolomics and choroid plexus transcriptomics to reveal an additional set of biologically relevant pathways. Pathway analysis combines pathway overrepresentation and topology analysis to evaluate which pathways are represented, as well as the likelihood of these being perturbed due to the experimental conditions. As such, pathway analysis relies heavily on the position (topology) of certain metabolites, or genes, within a metabolic pathway as well as the overall enrichment (overrepresentation) of these; i.e. that the gene or metabolite is present within the data set. Among the high-impact pathways were the glycerolipid and glycerophospholipid metabolism, pathways linked to energy consumption, in addition to the nicotinamide and nicotinate metabolism pathway, the latter regulating the amount of nicotinamide adenine nucleotide (NAD^+^), which is involved in regulation of CLOCK genes (57). Of note, the citric acid cycle and glycolysis/gluconeogenesis were impacted by the diurnal fluctuations in the joint pathway analysis, which aligned with the diurnal fluctuation of CSF lactate content, and with the dark phase up-regulation of genes involved in the citric acid cycle and glycolysis. These pathways are linked to energy metabolism in the brain, with glycolytic metabolism associated with the awake state during the dark phase (58, 59). This shift in choroid plexus metabolic pathways may underlie a functional shift in tissue function and thus CSF composition (19). A full metabolic analysis may encompass proteomics and lipidomics, in addition to the metabolomics and transcriptomics here combined. With these factors lacking, we may miss important regulatory compounds and/or parts of the biological pathways, which reduces the statistical impact and thus represents a limitation to the study. An additional limitation is the chosen two time points. These may not represent peak timing for all or some metabolites and transcripts, and one could obtain different results, had other, or even more, time points been employed.

In conclusion, our data suggest diurnal modulation of choroid plexus gene expression as well as CSF metabolite composition, which, alone or via joint pathways, may govern the diurnal modulation in CSF dynamics. Once delineated, these pathways could serve as potential pharmaceutical targets for modification of the CSF secretion rate, possibly to promote the dark phase increase in CSF secretion that may support nightly increase in metabolite clearance from the brain.

## Materials and methods

### Sample collection from experimental rats

Male Sprague–Dawley rats, 9 weeks old (Janvier), housed in temperature-controlled facilities with a 12:12-h light cycle, were employed for the study. The rats had access to water and food ad libitum and were randomly assigned to experimental groups. Samples were collected during the light phase (8 h after light phase initiation, zeitgeber time 8 h) and in the dark phase (8 h after dark phase initiation, zeitgeber time 20 h). The time points were chosen according to Borjigin and Liu (60), where the serotonin and melatonin levels are fairly constant and with sufficient time interval from the serotonin surge taking place in the dark phase. These time points have been employed to determine the diurnal fluctuation in rat CSF secretion rate and ICP (9). The dark phase samples were obtained in complete darkness except for a red LED lamp for dark adaptive lighting (OcuScience) occasionally lit to ensure that light exposure was minimal during the experimental procedures. Rats were anesthetized with 6 mg/ml xylazine + 60 mg/ml ketamine (ScanVet) in sterile water (0.17 ml/100 g of body weight [intraperitoneal injection]) and redosed with half ketamine dose as required to sustain anesthesia. CSF samples were collected under anesthesia in mechanically ventilated rats, where a tracheotomy was performed to control ventilation using the VentElite system (Harvard Apparatus) by 0.9 l/min humidified air mixed with 0.1 l/min O_2_ adjusted with approximately 3 ml/breath, 80 breath/min, a positive end-expiratory pressure (PEEP) at 2 cm, and 10% sigh for a ∼400-g rat. Body temperature during the experiment was maintained at 37°C by a homeothermic monitoring system (Harvard Apparatus). A cisterna magna puncture was performed with a glass capillary (30-0067, Harvard Apparatus pulled by a Brown Micropipette puller, Model P-97, Sutter Instruments), and approximately 100 µl CSF was collected and centrifuged (2,000 *g* for 10 min at 4°C). The supernatant was transferred to a new microtube (72-730-006, Sarstedt AG & Co) and immediately placed on dry ice prior to placement at −80°C until analysis. Rat choroid plexus (lateral and fourth) was acutely isolated following sacrificing of the anesthetized rat and stored in RNAlater (Sigma) at −80°C until analysis. All animal experimental works conformed to the legislations for animal protection and care in the European Community Council Directive (2010/63/EU) and were approved by the Danish Animal Experiments Inspectorate (license no. 2016-15-0201-00944).

### Metabolomics analysis of rat CSF

Sample analysis (*n* = 23, 11 light phase and 12 dark phase) was carried out by MS-Omics ApS (Vedbæk, Denmark) using an untargeted metabolomic approach. Semipolar metabolite separation was achieved with a slightly modified version of the protocol described by Doneanu et al. (61). The reverse phase analysis was carried out in a randomized order using a Ultra-High-Performance Liquid Chromatography (UHPLC) system (Vanquish, Thermo Fisher Scientific) equipped with a Water Acquity HSS T3 (2.1 × 150 mm, 1.7 µm) column, coupled with a high-resolution quadrupole-orbitrap mass spectrometer (Thermo Q Exactive HF, Thermo Fisher Scientific). The ionization was achieved with an electrospray ionization interface operated in positive and negative ionization modes. A series of pooled sample QCs were analyzed in MS/MS fragmentation mode under iterative exclusion for the identification (ID) of compounds. Untargeted and semitargeted data processing was performed using a custom workflow in Compound Discoverer 3.1 (Thermo Fisher Scientific), Skyline 22.1 (MacCoss Lab Software), and MATLAB 2021b (MathWorks). Feature extraction and grouping were performed without retention time alignment or normalization. Annotations are based on predicted sum formula and accurate mass matching to the HMDB version 4.0, spectral library matching to mzCloud (Thermo Fisher Scientific), as well as retention time and spectral matching to the MS-Omics in-house metabolite library (2021). ID of compounds was performed at four levels: level 1, ID by retention times (compared against in-house authentic standards), accurate mass (with an accepted deviation of 3 ppm), and MS/MS spectra; level 2a, ID by retention times (compared against in-house authentic standards) and accurate mass (with an accepted deviation of 3 ppm); Level 2b, ID by accurate mass (with an accepted deviation of 3 ppm) and MS/MS spectra; and level 3, ID by accurate mass alone (with an accepted deviation of 3 ppm). Out of the 987 compounds present in the CSF samples, 136 were identified as metabolites on levels 1, 2a, and 2b, with 10 metabolites subsequently excluded as the molecular weight and molecular ID did not match the HMDB. Main class enrichment analysis was generated using MetaboAnalyst (62) and performed on the CSF metabolites detected in all samples (light–dark phases). To assess light–dark phase variations of metabolites, we calculated the descriptive power of each compound as the ratio between the standard deviations obtained from the experimental sample and the quality control samples. Thirty metabolites had a descriptive power below 2.5 and were therefore assigned to not fluctuate with the light–dark phases. The metabolomic data were tested using PCA with GraphPad Prism (GraphPad Prism version 9.5.1 for Windows, GraphPad Software) prior to outlier testing with Smirnov–Grubbs test. Light–dark phase metabolite fluctuation was determined by normalizing peak values for each sample to the geometric mean of the quality control samples and employing a FDR of <0.10 according to the Benjamini–Hochberg method (63, 64). Differential presence of CSF metabolites is presented as the log_2_-transformed fold change from light to dark phase, with negative values indicating a higher value in the light phase and positive values indicating higher values in the dark phase.

### RNA-seq analysis of rat choroid plexus

RNA extraction from the excised choroid plexus (*n* = 12, 6 from each phase) and library preparation was performed by Novogene Company Limited with NEB Next Ultra RNA Library Prep Kit (NEB) prior to RNA sequencing (paired-end 150 bp, with 12-Gb output) on an Illumina NovaSeq 6000 (Illumina). Raw data are available at the National Center for Biotechnology Information (NCBI) Gene Expression Omnibus (GEO) database (GSE228866). Quality control and adapter removal were done by Novogene. The 150 base paired-end reads were mapped to rat reference genome Rnor_6.0.104 (*Rattus norvegicus*) using Spliced Transcripts Alignment to a Reference (STAR) RNA-seq aligner (v. 2.7.9a) (65). The mapped alignment generated by STAR was normalized to TPM with RSEM (v. 1.3.3) (66, 67), and raw counts from STAR GeneCounts were used for differential expression analysis using DEseq2 (68). Gene information was collected from Ensembl BioMart (69). PCA plot was conducted with GraphPad Prism (GraphPad Prism version 9.5.1 for Windows, GraphPad Software) prior to outlier testing with Smirnov–Grubbs test. Differential expression was determined based on the standard procedure of DEseq2 analysis with a FDR of <0.05, Benjamini–Hochberg method (70). Differential expression of choroid plexus genes is presented as the log_2_-transformed fold change from light to dark phase, with negative values indicating a higher value in the light phase and positive values indicating higher values in the dark phase. GO enrichment analyses were obtained with the PANTHER database (71) with a combination of UniProt ID (72) and gene symbols, classifying the protein class and molecular function of each differentially expressed gene. Circadian clock system genes present within the data set were identified using the data set as input to the PANTHER database to classify GO enrichment pathways. Circadian rhythm–associated genes were identified with a search for “circadian” among protein and pathway descriptors on genecards.org. The count table with TPM normalization was utilized for tables and bar charts. Compartment scores for protein-coding genes were calculated (73) to ascribe these to either the plasma membrane or the mitochondria with scores above 2.4 and no other compartment with a higher, or equal, score. A small excerpt of RNA-seq data obtained from this choroid plexus tissue (transcripts encoding eight membrane transporters and one kinase) is included in Steffensen et al. (9). Scripts and program parameters can be found online (https://github.com/Sorennorge/Day_night_transcriptomics_and_CSF_metabolomics).

### Pathway analysis

Metabolic pathways were identified with pathway, and joint pathway, analysis employing MetaboAnalyst (62), using “HMDB IDs” for metabolites and “gene symbols” for genes. This analysis tool combines pathway enrichment (hypergeometric overrepresentation) and topology analysis (relative-betweenness centrality) to identify the significance (enrichment) and impact factor (summed impact of metabolites/genes present according to topology) (74). Both analyses were targeted for *R. norvegicus* and parameters were set to hypergeometric, relative-betweenness centrality and combined *P* value. Significant pathways were defined as having a FDR < 0.10, with the perturbation determined according to the combination of significance and impact factor (74, 75). For pathway analyses of diurnal modulation, the log_2_ fold change was manually set to 0 for metabolites and genes not meeting the requirements for differential presence/expression. All pathways were ascribed with the subclass identified within the Kyoto Encyclopedia of Genes and Genomes (KEGG) Database.

## Supplementary material

Supplementary material is available at *PNAS Nexus* online.

## Supplementary Material

pgad262_Supplementary_Data

## Data Availability

The raw data set for metabolomics data is accessible through the NIH Common Fund’s National Metabolomics Data Repository (NMDR, supported by NIH grant, U01-DK097430 and U2C-DK119886) website, the Metabolomics Workbench, https://www.metabolomicsworkbench.org, study ID ST002772 (http://dx.doi.org/10.21228/M86X4K). Raw RNA-seq data are available at the NCBI GEO database with accession number: GSE228866 (https://www.ncbi.nlm.nih.gov/geo/query/acc.cgi?acc=GSE228866). Quantified metabolomic data are available in Table S1 (LC-MS data), and scripts and data analysis can be found here: https://github.com/Sorennorge/Day_night_transcriptomics_and_CSF_metabolomics.
