# Supplementary material for: Day–night fluctuations in choroid plexus transcriptomics and cerebrospinal fluid metabolomics
Source: PNAS Nexus. 2023 Aug 10;2(8):pgad262. doi: 10.1093/pnasnexus/pgad262 (PMC10443925; doi:10.1093/pnasnexus/pgad262)
Supplement: pgad262_Supplementary_Data [file pgad262_supplementary_data.zip › PNASNEXUS-PNASNEXUS-2023-00357R-s02.pdf]

**Supporting Information for**
Day-night fluctuations in choroid plexus transcriptomics and cerebrospinal fluid metabolomics

Beatriche Louise Edelbo, Søren Norge Andreassen, Annette Buur Steffensen and Nanna MacAulay

Nanna MacAulay
Email: macaulay@sund.ku.dk

**This PDF file includes:**

Figures S1 to S4

**Other supporting materials for this manuscript include the following:**

SIAppendix_TableS1 to S13

# Supplemental figures

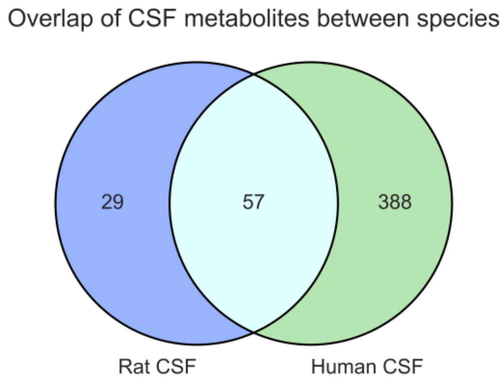


**Figure S1. Comparison of human and rat CSF metabolome**. Overlap of CSF metabolites in samples from rat and humans. Rat CSF metabolites was based on LC-MS on CSF collected from 11 rats in the light phase and 12 rats in the dark phase, excluding metabolites with a descriptive power above 2.5 (*SI Appendix,* Table S1). Human CSF metabolites included were all recognized as CSF specific within HMDB. Overlapping metabolites are listed in Table S1 (*SI Appendix*).

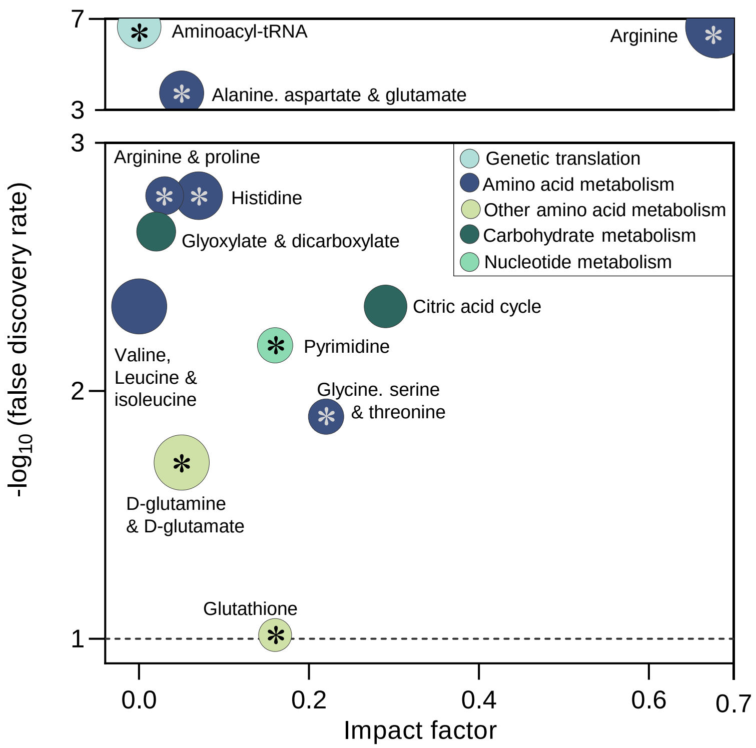


**Figure S2. Pathway analysis of the diurnally regulated CSF metabolome**. Pathways are organized according to the collected impact of the detected pathway-specific metabolites (x-axis) against the probability of the metabolite composition being a function of that particular pathway (y-axis). Sphere color specifies the subclass of each pathway (see inset), sphere area indicates the number of metabolites detected compared to the expected number (fold enrichment, *SI Appendix*, Table S3). The dashed line indicates cut-off for pathway significance (at a false discovery rate < 0.10). Data are based on CSF collected from 11 rats in the light phase and 12 rats in the dark phase. The pathways that contain metabolites that fluctuate with the light-dark phases (see Fig. 2B) are depicted with an asterisk.

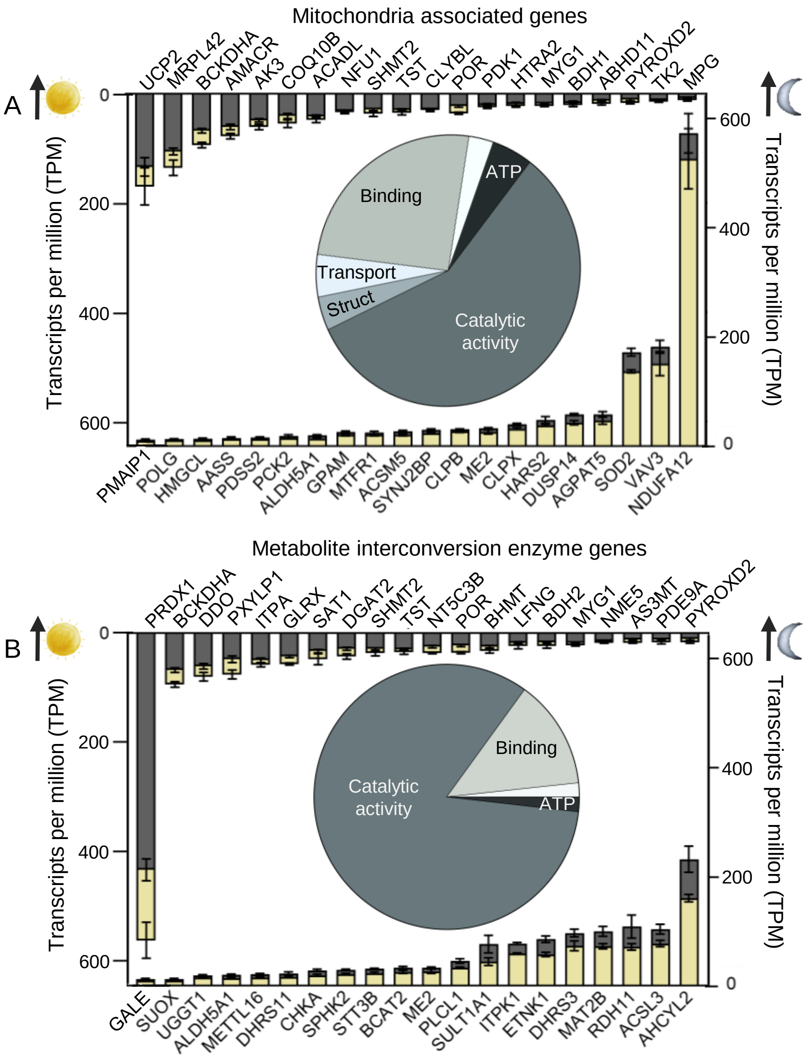


**Figure S3. Characterization of the diurnally regulated choroid plexus mitochondrial compartment and metabolite interconversion enzymes transcriptome.** Choroid plexus gene expression of the 20 **A.** mitochondria-associated genes, **B.** metabolite interconversion enzyme genes, with the most pronounced shifts between the dark-light phases and a summed TPM > 20 (dark phase gene expression illustrated in grey and light phase gene expression illustrated in yellow). Data include genes that are significantly modulated between the two phases (false discovery rate < 0.05), are shown as mean TPM ± SD and based on n = 6 choroid plexus obtained in either phase. Inset; the molecular function of differentially expressed genes with a molecular function assigned (**A.** excluding 49 genes (35%); **B.** excluding 70 genes (24%)). Categories amounting to ≤ 2% are collected in the non-labelled white pie (**A.** molecular adaptor activity, cytoskeletal motor activity; **B.** Molecular function regulator, transcription regulator activity, transporter activity). ATP; ATP-dependent activity. Transport; transporter activity, Struct; structural molecule activity.

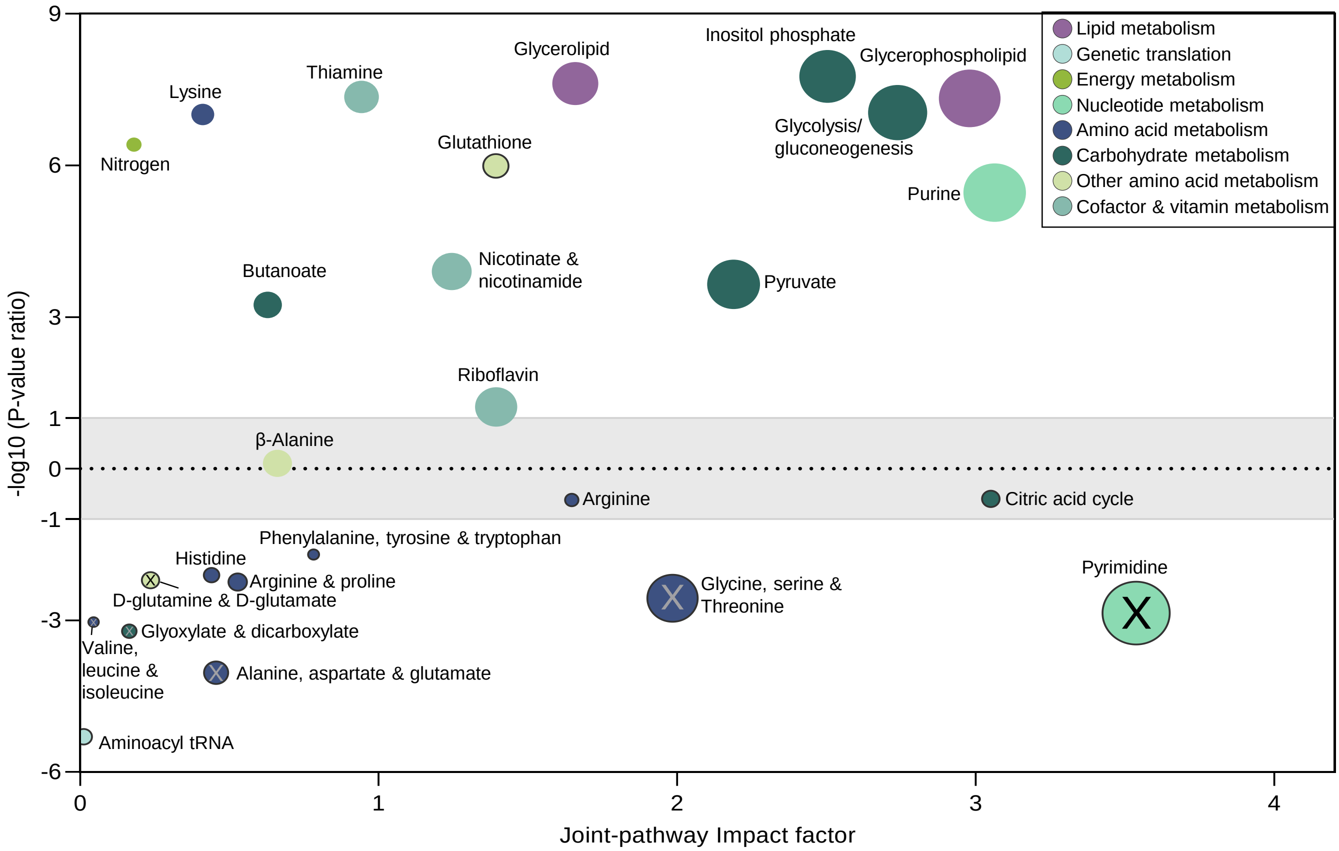


**Figure S4. Light-dark phase modulation of the significant CSF biological pathways occurring with inclusion of the choroid plexus transcriptome**. Comparison between the P-value and impact factor for pathways identified prior and subsequent to inclusion of choroid plexus transcriptome in the pathway analysis. Pathways are organized according to the collected impact of joint pathway-specific metabolites detected (x-axis) against the P value ratio (P value obtained from the joint pathway analysis (Fig. 4) divided by the P value obtained from the pathway analysis of the diurnally regulated metabolomics (Fig. S2)). Sphere color specifies the subclass of each pathway (see inset). The sphere area signifies the number of metabolites detected compared to the expected number (fold enrichment, *SI Appendix*, Table S12). Pathways above the grey band centering the zero value increased in significance when dark-light phase transcript fluctuations were included in the analysis, whereas those below reduced in significance. X depicts the biological pathways that are no longer significant in the CSF after inclusion of the choroid plexus transcriptomics (see *SI Appendix*, Table S12). Circles without a border are significantly enriched only upon inclusion of the transcriptome. The dashed line indicates cut-off for pathway significance with a false discovery rate < 0.10. Data are based on CSF samples from n = 11 rats (light phase) and n = 12 rats (dark phase) combined with choroid plexus transcriptomics of n = 6 rats in each phase.
